# Genetic Labeling of Neuronal Subsets through Enhancer Trapping in Mice

**DOI:** 10.1371/journal.pone.0038593

**Published:** 2012-06-07

**Authors:** Wolfgang Kelsch, Alberto Stolfi, Carlos Lois

**Affiliations:** 1 Bernstein Center for Computational Neuroscience, CIMH, Medical Faculty Mannheim, University Heidelberg, Heidelberg, Germany; 2 Center for Developmental Genetics, Department of Biology, New York University, New York, New York, United States of America; 3 Department of Neurobiology, University of Massachusetts Medical School, Worcester, Massachusetts, United States of America; Columbia University, United States of America

## Abstract

The ability to label, visualize, and manipulate subsets of neurons is critical for elucidating the structure and function of individual cell types in the brain. Enhancer trapping has proved extremely useful for the genetic manipulation of selective cell types in Drosophila. We have developed an enhancer trap strategy in mammals by generating transgenic mice with lentiviral vectors carrying single-copy enhancer-detector probes encoding either the marker gene lacZ or Cre recombinase. This transgenic strategy allowed us to genetically identify a wide variety of neuronal subpopulations in distinct brain regions. Enhancer detection by lentiviral transgenesis could thus provide a complementary method for generating transgenic mouse libraries for the genetic labeling and manipulation of neuronal subsets.

## Introduction

**Figure 1:**
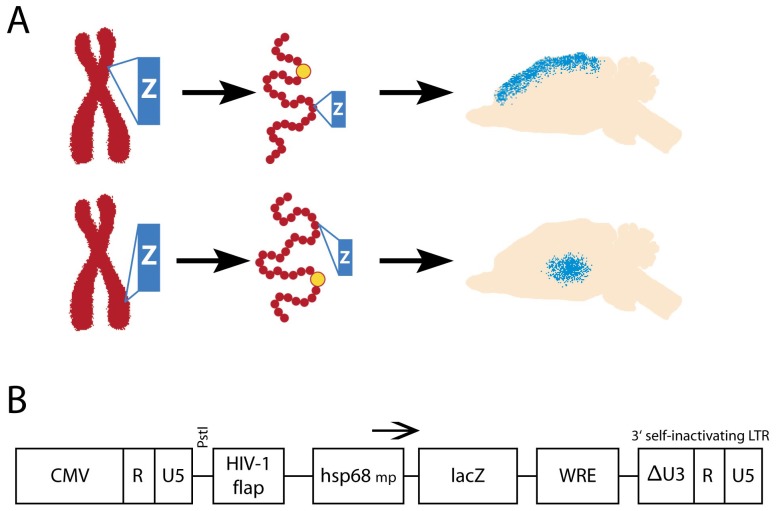
Generation of transgenic mice carrying a nlacZ enhancer probe under control of a hsp68 minimal promoter. Enhancer probes integrate into different sites in the genome. Depending on the site of integration the interaction of the introduced minimal promoter and enhancer elements of the genome results in restricted expression of the transgene.

Mammalian brains contain a bewildering variety of different classes of neurons. Interestingly, it is still not known how many different neuronal types exist in the mouse or rat brain, the most commonly used laboratory animals. The number of distinct neuronal types steadily increases as new analyses and techniques better differentiate those using combined anatomical, electrophysiological, and genetic criteria [1], [2], [3]. The ability to identify specific neuronal types will be critical for understanding their contribution towards brain function and behavior [4]. Moreover, to investigate the function of genetically defined subsets of cells, it is necessary not only to visualize them, but also to selectively manipulate their gene expression. Transgenic animals expressing fluorescent markers in neuronal subsets [5] have proven very useful for *in vivo* imaging and electrophysiology. However, there is currently only a limited number of mouse lines that express visible markers in subsets of neurons [5], [6], and only a few lines can be used for selective gene manipulation in these neuronal populations [7]. We have developed a new mouse transgenic strategy in which expression of the recombinase Cre depends on enhancer detection, with the goal of creating libraries of transgenic mice with the ability to visualize and manipulate genes in selective subsets of neurons. To achieve this goal, we used lentiviral transgenesis to deliver enhancer-detection probes into single-cell mouse embryos. Lentiviruses integrate preferentially into gene-rich regions of the genome [8], thereby increasing the chance that a transgene insertion will be activated by nearby enhancers. The strategy of enhancer detection relies on a gene of choice present within a transgenic probe whose transcription depends on where the probe integrates in the genome. In eukaryotic cells, gene expression depends on the presence of cis-DNA sequences that regulate the rate of transcription of the gene and transcription factors that recognize them [9]. The two major classes of DNA *cis*-regulatory transcriptional elements are long-range and short-range elements. Short-range regulatory elements, called promoters, are located immediately upstream of the gene they regulate. In contrast, long-range regulatory elements can be located either within introns, upstream or downstream of the transcription start site, sometimes up to hundreds of kilobases away from the gene whose activity they regulate [10]. These distant regulatory elements can either have a positive or a negative effect on transcription, and they are designated as enhancers or silencers, respectively. In line with the traditional nomenclature used in enhancer trapping, here we will refer to both of these elements as enhancers for simplicity. By themselves, neither enhancers nor promoters are sufficient to drive transcription. The functional expression of genes requires the combined activity of enhancers and promoters, situated in a specific configuration with respect to the gene they regulate. The strategy of enhancer detection is based on the requirement of promoters to be activated by enhancers to achieve expression of the genes they regulate [11] (Figure 1). We have engineered lentiviral vectors encoding enhancer detection probes that allowed efficient generation of transgenic mice selectively expressing Cre under the influence of enhancers located in the vicinity of the chromosomal integration site. We demonstrated the utility of these transgenic mice for the identification of specific neuronal classes and for their electrophysiological characterization. In addition, these mice can be used to perform Cre-mediated gene manipulation in selective neuronal types. Our results demonstrate that enhancer detection, a technique that has proven extremely valuable to genetically identify and manipulate cells in invertebrates, could provide a complementary approach to genetically dissection of the neuronal diversity of the mouse brain.

## Results

### Efficient Enhancer Trapping with a *lacZ* Reporter

**Figure 2:**
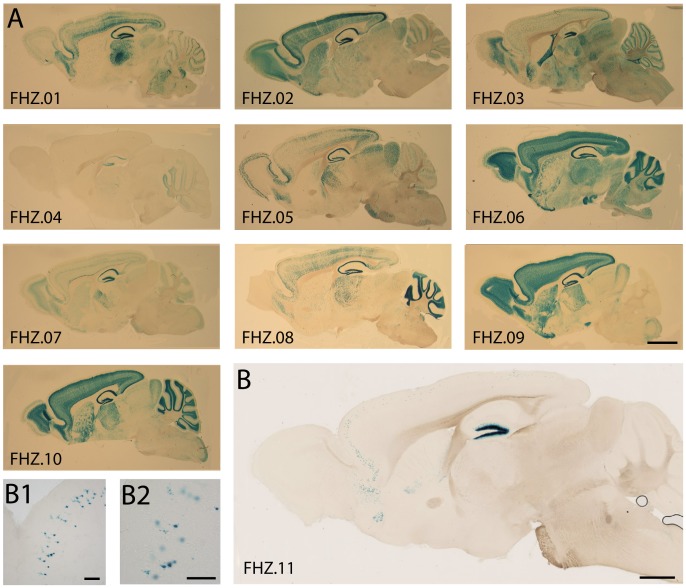
Labeling of subsets of neurons in hsp68-nlacZ lines. (A), (B) Sagittal sections of mice carrying a single copy of the hsp68-nlacZ transgene. A large variety of subsets of neurons was labeled in eleven independent mouse lines labeled FHZ.01 to FHZ.11 ((A) bar = 1.5 mm, (B) bar = 1 mm). (B1), (B2) Higher magnification views show a population of labeled cortical neurons in the frontal cortex of the brain shown in B (both bar = 100 um).

**Figure 3:**
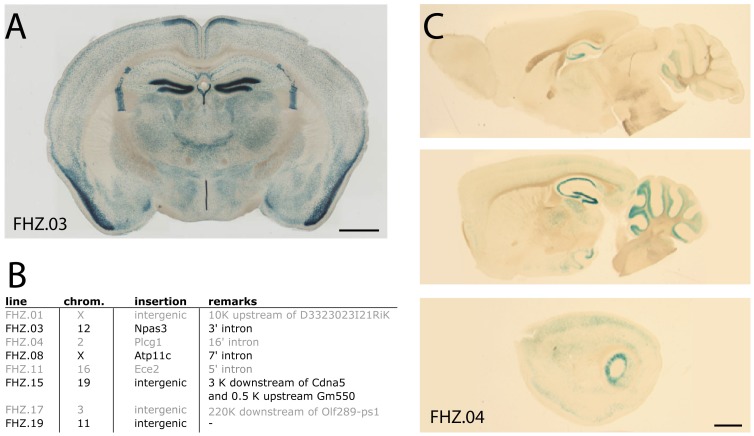
Labeling of subsets of neurons in different hsp68-nlacZ lines. (A) Coronal section of a mouse carrying a single copy of the hsp68-nlacZ transgene that integrated into the coding region of the Npas3 gene (bar = 1 mm). (B) The table shows the genomic insertion site of eight mouse lines carrying a single copy of the hsp68-nlacZ transgene. (C) Sagittal sections of a mouse carrying a single copy of the hsp68-nlacZ transgene that integrated into the coding region of the Phospholipase C-gamma1 gene. Medial to lateral sections of the brain reveal a medio-lateral expression gradient both in the hippocampus and cerebellum (bar = 1mm).

In order to test the applicability of enhancer detection by lentiviral vectors, we first generated a lentiviral enhancer probe containing the minimal promoter of the human heat-shock gene 68 (hsp68), which by itself has a very low basal level of activity and must be activated by an enhancer on the same chromosome to achieve expression of a reporter gene [12]. We then generated transgenic mice by infecting single-cell mouse embryos with a recombinant lentiviral vector carrying the hsp68 minimal promoter controlling the expression of the nlacZ gene, which contains a nuclear localization sequence. Treated embryos were then returned to the uterus of surrogate mothers to complete development. Transgenic founders were bred with wild type animals to obtain a single copy of an insertion in the offspring. To maximize the chances of obtaining animals in which single-copy transgenes could be generated within one breeding cycle, we titrated the lentiviral vector in our procedure so that we obtained 1–4 insertions per founder animal that could be separated by breeding over 1–2 generations. Most animals (23 out of 25 lines) carrying a single copy insertion displayed distinct expression patterns in the brain when assayed by lacZ histochemistry. The pattern of nlacZ-expressing cells in the different transgenic lines ranged from near-ubiquitous to restricted to, for example, specific layers in the cortex, subregions of the hippocampus, and medio-lateral gradients within the same structure (Figure 2). We cloned the insertion site of eight different lines by ligation-mediated PCR. As expected from previously published works [8], four out eight insertions were located within introns, and the remaining four insertions were located either upstream or downstream of the genes’ coding region (Figure 3).

The patterns of lacZ expression from the transgenic animals we analyzed did not faithfully match that of the endogenous genes into which it integrated. For example, transgenic line (FHZ.03, Figure 3) had an insertion into the third intron of Npas3, a gene mutated in rare forms of familial schizophrenia [13]. The expression of the endogenous NPAS3 gene is broadly expressed in the adult mouse brain [13]. In contrast the FHZ.03 transgenic line displayed a more restricted pattern than that of the endogenous Npas3 with layer-specific expression in the hippocampal dentate gyrus, and in the retrosplenial and piriform cortices. The interaction between minimal promoters and enhancers is specific, such that not all minimal promoters will interact with all enhancers. Therefore, it is anticipated that in enhancer detection strategies the expression pattern of the reporter gene present in the detector probe may not faithfully reproduce the expression pattern of the endogenous gene where it integrates [10], [14]. However, the appearance of patterns of expression resulting from enhancer detection in transgenic animals that do not faithfully reproduce those of endogenous genes could be advantageous in many situations since some of these patterns could label selective populations of neurons, as shown in Figure 2 and 3).

### Cre-based Transgenics with a GFP Reporter

The initial experiments with the nlacZ probe confirmed the validity of the strategy of genetic labeling of neuronal types by enhancer detection. Although visible markers such as lacZ or GFP are useful for the visualization of neurons, they cannot be used for manipulation of gene expression. Selective gene manipulation can be obtained using a Cre/loxP-system where expression of the Cre enzyme in subsets of neurons regulates the expression of a ‘floxed’ gene. Accordingly, we proceeded to generate mice with an enhancer detector probe expressing Cre under the control of the hsp68 minimal promoter.These founder transgenic animals were then bred to single copy insertion by crossing them with the cre-dependent GFP reporter line (Z/EG) [15], that allows detection of recombination in neurons by expression of cytoplasmic GFP. However, when we examined the brains of these mice, we observed that in most cases, the recombination patterns were too broad in the brain, and therefore not useful for most experiments (data not shown). This near-ubiquitous pattern of expression results from the fact that once Cre recombines a loxP cassette in a given cell, all the progeny of these cells will inherit the recombined loxP allele (‘prospective’ labeling). Thus, if the hsp68-cre cassette leads to expression in some cells during early embryonic development, this would lead to a very high number of ‘floxed’ cells in the brain. Consistent with this hypothesis, we observed that animals with broad GFP expression (due to loxP recombination in the Z/EG mice) showed sparse or no persistent Cre expression in adulthood (data not shown).

**Figure 4:**
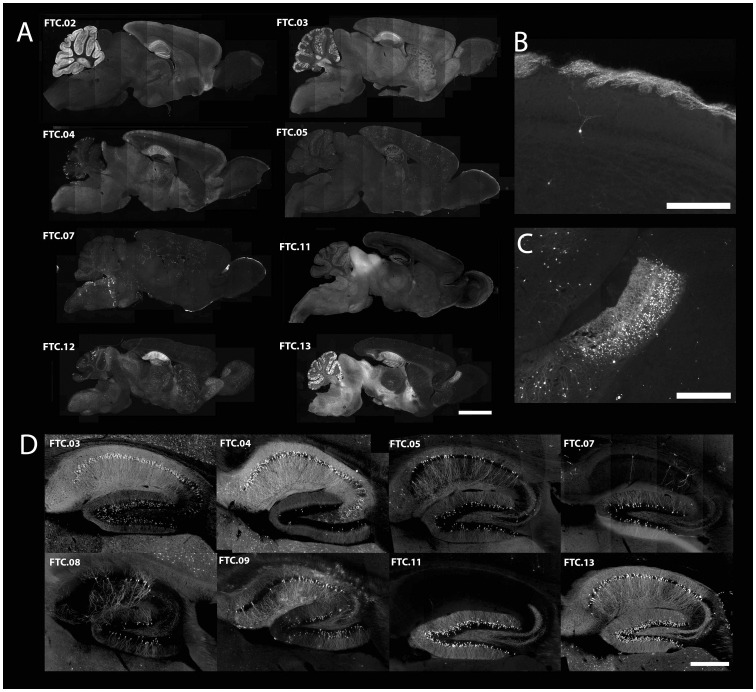
Labeling of subsets of neurons in thy1mp-cre lines. (A) Sagittal sections of mice carrying a single copy of the thy1mp-cre transgene (lines named FTC.01-FTC.13). A large variety of subsets of neurons were labeled in eight independent mouse lines shown here (bar = 2.5mm). (B) Labeling of olfactory receptor neurons (FTC.07) (bar = 250 um). (C) Labeling of granule cell neurons in the accessory olfactory bulb (FTC.13) (bar = 500um). (D) Sagittal sections of the dorsal hippocampal formation of eight different thy1mp-cre lines reveal recombination restricted to subregions of the hippocampus (CA1, CA3 or dentate gyrus) (bar = 500 um).

In order to bias the specificity of Cre expression to neurons, and to reduce the appearance of ubiquitous patterns of recombination, we generated a lentiviral enhancer detection probe carrying cre under the control of the minimal promoter of the thy-1.2 gene, consisting of 310 base pairs upstream from its transcription start site (thy1mp-cre) [16]. The thy-1.2 gene is preferentially expressed in projection neurons in the mouse brain, and its minimal promoter may contain elements that could restrict its activity to neurons [17], [18]. Founder transgenic animals carrying 1–4 insertions of thy1mp-cre were bred to the Cre-dependent GFP reporter lines (Z/EG) to obtain lines with single copy insertions of the enhancer detection probe. We observed a large diversity of restricted recombination patterns in 16 out of 20 lines (Figure 4A). The pattern of recombination was reproducible among animals with the same single insertion of the enhancer probe (Figure S1). In the hippocampus, for example, we observed that recombination patterns were frequently restricted to specific substructures (CA1, CA2 or dentate gyrus, Figure 4D). We also observed transgenic lines that labeled specific neuronal types, such as of olfactory receptor neurons (FTC.07, Figure 4B) and granule cells of the accessory olfactory bulb (FTC.13, Figure 4C). In certain cortical areas, we observed that recombination patterns were restricted to specific layers (Figure 4A).

### Variability of Functional Properties in Neuronal Subsets

**Figure 5:**
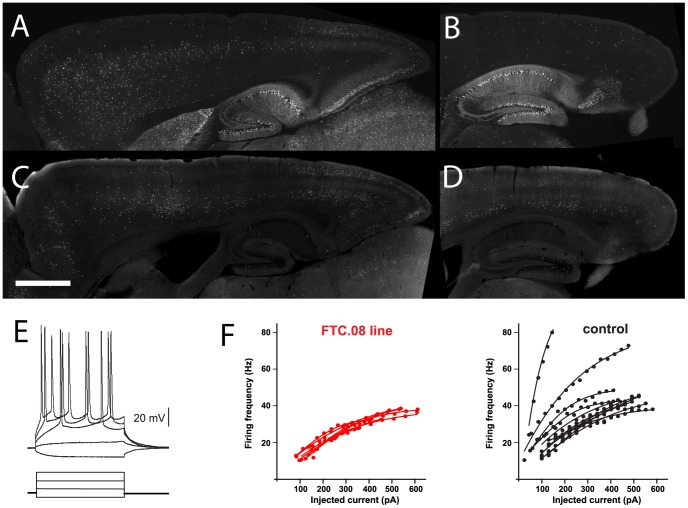
Variability of functional properties in neuronal subsets in thy1mp-cre lines. (A)-(D) Sagittal sections of thy1mp-cre lines (FTC.13 ((A), (B)) and FTC.08 ((C), (D)) reveal changes in the layer-specific expression in the frontal versus occipital cortical areas. In addition, in the occipital cortex the layer-specific expression changed from medial ((A), (C) corresponding to retrosplenial cortex) to lateral ((B), (D) corresponding to visual cortex) (bar = 1 mm). (E) Whole-cell recordings from visual cortex layer 2/3 of thy1mp-cre line FTC.08 were performed from GFP positive and GFP negative neurons in the same animals (n = 7 and 11, respectively). The relationship between the amount of injected current and the frequency of induced action potential was plotted for each recorded cell. GFP positive layer 2/3 neurons from FTC.08 had a smaller variability in their frequency-current relationship than GFP negative layer 2/3 pyramidal neurons in the same animals. (F) shows the adaptation index for GFP positive and GFP negative control layer 2/3 pyramidal neurons from line FTC.08 (n = 7, 11, respectively). The adaptation index indicates the ratio of the second interspike interval (ISI) over the last ISI of a series of pulses (200 ms).

In several independent transgenic lines, cells with layer-specific recombination in the cortex had the morphology of pyramidal neurons. To examine whether these layer-specific transgenic lines contained subsets of neurons defined by specific electrophysiological properties, we selected the transgenic line FTC.08 for fluorescence-guided whole-cell recordings that had GFP^+^ neurons in layer 2/3 in the visual cortex (V1 area, Figure 5A-D). To determine the homogeneity of the electrophysiological properties of the GFP^+^ cells, we compared them to GFP^−^ control neurons in layer 2/3 in the same animals. We observed that in this transgenic line the variability in some of the electrical properties of GFP^+^ cells, such as frequency–current relationship or adaptation index, was smaller in GFP^+^ than in GFP^−^ neurons of the same layer (Figure 5E, F, see also Figure S2 for additional data). These observations underscore the usefulness of the enhancer trap approach to identify neuronal populations with defined properties.

## Discussion

We have developed a genetic approach to efficiently generate large numbers of transgenic mouse lines that selectively express Cre in subsets of neurons based on enhancer detection. Such Cre mouse lines can be used for fluorescence-guided recordings, gene expression profiling, *in vivo* imaging, and gene manipulation. The flexibility of the Cre-based site-specific recombination system allows cell-type specific gene ablation [19], opto-genetic control of neuronal activity [20] and trans-synaptic tracing of connections of genetically defined neurons [21] among other available techniques. This flexible system could be particular useful in cellular and system neuroscience where multiple cell-types contribute to the function of the system. Creating these mouse lines using the mammalian enhancer trap strategy complements existing approaches that aim to provide neuronal-type specific expression in transgenic animals. Transgenic mice generated with bacterial artificial chromosomes (BACs) that express GFP or Cre under the transcriptional regulatory elements of candidate genes of interest, e.g. calcium binding proteins [6], [7], are well suited to characterize neuronal types that express known genes. In contrast, the enhancer trap technique promises to yield a larger number of recombination patterns as it benefits from the random integration of the probe into different genomic loci, and subsequent interaction between the promoter present in the probe and the enhancer elements present in the genomic DNA. This random genomic insertion pattern could be an advantage to address some specific problems in neurobiology, such as the classification of interneuron types in the brain. Traditionally, classes of interneurons have been defined by the expression of genes such as parvalbumin, calbindin, or cholecystokinin [2]. Transgenic mice have been generated expressing marker genes under the regulatory elements that control the transcription of these proteins, but the labeled neurons can be heterogeneous both in their morphology and electrophysiological properties [2]. This observation suggests that the expression of single genes could not be used to identify functionally distinct neuronal types. Thus, it is likely that the classification of neurons will require an intersectional strategy that is requiring the sharing of at least two different characteristics, for example, such as expression of a particular calcium-binding protein and a specific type of ion channel. In the enhancer detection strategy, the pattern of expression depends on the interaction between the minimal promoter present in the probe and the enhancer element present in the genomic insertion site. In our nlacZ lines, the active expression of the marker gene in the probe can be directly detected (without the prospective labeling that occurs after Cre-recombination). We observed that in three of them that had insertions inside of introns (into the genes Npas3, Atp11c and PLC-gamma), the pattern of expression of the nlacZ gene overlapped, but was more restricted than the endogenous genes into which it inserted. The transcription of the endogenous gene is regulated by the interaction between multiple enhancers with its own minimal promoter. It is known that the interaction between enhancers and promoters shows some specificity, with some enhancers interacting with some promoters but not others. In our transgenic animals, transcription of the marker genes (lacZ or cre) depends on the interaction between the minimal promoters present in the transgenic probe (hsp68 or thy1mp) and some, but most likely not all, the enhancers neighboring the insertion site. Thus, it is expected that the pattern of expression originating from the enhancer detector probe will not recapitulate faithfully the expression pattern of the endogenous gene close to its insertion site, but instead, it would be biased by the ability of the minimal promoters to interact with some of the enhancers. To further restrict the subset of neurons labeled with this approach, it would be possible to take advantage of enhancer detector probes with biased minimal promoters, which will further limit the interactions between the probe promoter and the genomic enhancers. For example, a probe containing a minimal promoter derived from the gad67 gene could yield transgenic lines biased towards subsets within this population of interneurons.

The technique of enhancer detection has been applied in experimental animals such as *Drosophila* with great success [11], [22]. Although a few works have described the production of transgenic mice carrying enhancer detector probes, technical limitations have hampered the use of this strategy [11], [23]. First, transgenes introduced by pronuclear injection in mice integrate as head-to-tail tandem arrays of multiple copies of the same construct in a single chromosomal location and are prone to epigenetic effects such as repeat induced silencing [24]. Second, the application of enhancer detection to a particular animal species requires the ability to efficiently introduce the enhancer probe into the genome of that animal, and previously available techniques such as gene targeting in embryonic stem cells and pronuclear injection are time-consuming, laborious and relatively inefficient [16], [25]. In contrast to the low success rate of approximately 10% when using these previous techniques, introducing genes into early mouse embryos via recombinant lentiviral vectors yields more than 80% transgenic animals and therefore can easily be scaled up for high throughput screening [26]. Furthermore, transgenes delivered by lentiviral vectors integrate as individual molecules in the chromosome, and thus are not subject to repeat-induced silencing. Our transgenic Cre lines provided reproducible recombination in the same subset of neurons within a mouse line carrying a single insertion, and each line revealed a distinct subset of cells. In the future, it should be possible to combine enhancer trapping with germline transposition [27] to facilitate the generation of transgenic lines with different integration sites that would label a high number of neuronal subsets.

It is likely that the genetic labeling of some of the neuronal populations identified by this strategy could not be achieved by transgenic techniques that reproduce the pattern of expression of endogenous genes. The strategy of enhancer trapping complements other existing technologies used to genetically identify neuronal types. Considering the potentially widespread application in cellular and system analysis with optical, electrophysiological, and genetic manipulations, this transgenic strategy could be used to provide an ‘off-the-shelf’ library of mouse lines specific for neuronal subsets. In the future, this transgenic approach could also be extended to other organisms of neurobiological interest such as rats and birds that are now easily accessible to transgenic manipulations [26], [28].

## Methods

### Generation of Enhancer Detector Constructs

The lentiviral vectors used for enhancer detection were based on our previously described lentiviral backbones that allow us to achieve high viral titers and high levels of expression [26], [29]. We introduced the human heat shock gene 68 (HSP68) minimal promoter consisting of 226 base pairs upstream from the transcription start site [12] into the FW backbone [26], [29]. This minimal promoter lacks enhancer activity and does not produce detectable transcription when introduced to cells as an episomal element. The reporter gene nlacZ, which contains a nuclear localization sequence, was placed downstream of the promoter. The same backbone was used to create the FTC lines, using the 310 base pair minimal promoter of the Thy-1.2 gene and a Cre:GFP fusion as the reporter gene (thy1mp-cre).

### Generation of Transgenic Mice with Enhancer Probes

Transgenic mice were produced as described before [26] by delivering concentrated lentiviral vectors into the perivitelline space. All procedures were approved by the local animal committees.

### Southern Blotting

All F0 transgenic mice were analyzed by Southern blot. Hsp68-nlacZ mice with multiple copies were crossed to CD-1 mice and subsequent generations also were examined by Southern to isolate single-copy insertions. F0 thy1mp-cre mice with 1–4 copies were crossed to homozygote Z/EG reporter mice, again with the F1 progeny analyzed by Southern to identify single-copy animals.

### Identification of Insertion Sites

In order to clone pieces of genomic DNA flanking the insertion site of the probe we performed ligation-mediated PCR (LM-PCR) from the genomic DNA of transgenic mice carrying single copies [30].

### Expression Patterns of Transgenic Mice

We analyzed the staining pattern of twenty-five animals carrying unique single-copy integrations of the hsp68-nlacZ proviral probe and twenty animals carrying unique integrations of the enhancer probe in fixed 50 µm thick sections. Mice were perfused transcardially with PBS and then 3% paraformaldehyde in PBS; brains were postfixed overnight at 4°C. Tissue was stained with an X-gal solution for animals expressing n*lacZ* or immunofluorescently stained with a rabbit polyclonal antibody raised against GFP (1:4000, Chemicon) or a monoclonal antibody raised against Cre (1:1000, Chemicon) and A488/555-conjugated secondary antibodies (Molecular Probes).

### Fluorescence-guided Recordings

Coronal 350 µm brain slices were prepared from the visual cortex of P45-P60 mice with a solution containing (in mM): 212 sucrose, 3 KCl, 1.25 NaH_2_PO_4_, 26 NaHCO_3_, 7 MgCl_2_, 10 glucose, and pH 7.3 at 4°C. Slices were recovered for 30 min at 35°C in ACSF containing (in mM): 125 NaCl, 2.5 KCl, 1.25 NaH_2_PO_4_, 26 NaHCO_3_, 1 MgCl_2_, 2 CaCl_2_, 20 glucose, and pH 7.3. Fluorescence-guided whole-cell recordings (Heka EPC-10) were performed at 22°C with pipette solution containing (in mM): 2 NaCl, 4 KCl, 130 K-gluconate, 10 HEPES, 0.2 EGTA, 4 ATP-Mg, 0.3 GFP-tris, 14 phosphocreatine, 0.02 Alexa555 hydrazide, and pH 7.3. Patch pipettes had 7 to 9 MOhm resistance. In the whole-cell configuration, either current steps of 5 ms (0.2–0.4 nA) were elicited to evoke single action potentials, or longer, incrementing current steps (200 ms) were applied to measure interspike intervals (ISI). The adaptation index indicates the ratio of the second ISI over the last ISI of a series of pulses (200 ms), and the frequency-current relationship was obtained plotting the action potential frequency during a 200 ms pulse against the injected current. Finally, overlay of GFP fluorescence and Alexa555 dye was confirmed. Biocytin fills (2–4 mg/ml intracellular solution, Sigma) were incubated with 1% avidin-biotinylatedhorseradish peroxidase complex containing 0.1% Triton X-100 (ABC-Elite) and then reacted using 3,3-diaminobenzidine(Pierce).

### Ethics Issues

No humans participants were involved in this study. Ethics approval by a committee was not necessary. The experiments involving animals were approved by the IACUC committee of the Massachusetts Institute of Technology.

## Supporting Information

Figure S1 **Consistent recombination patterns in transgenic mice carrying a Cre-expressing enhancer probe.** (A) Recombination patterns for GFP expression were consistent among animals with the same insertion site of thy1mp-cre. (A1)–(A3) show the dorsal hippocampi of three animals from the FTC.03 line. They shared the same expression pattern (bar = 500 µm). (B) In this transgenic line the persistent cre expression was consistent in three adult animals ((B1)-(B3)). In all animals the persistent cre expression in the adult was much smaller than the density of recombined GFP-positive neurons (bar = 500 um).
(TIF)

Figure S2 **Variability of functional properties in neuronal subsets in thy1mp-cre lines.** (A) Recording site in the visual cortex (V1 area) for GFP positive and GFP negative control layer 2/3 pyramidal neurons from line FTC.08. (B) Biocytin-filled GFP positive and GFP negative control layer 2/3 pyramidal neurons from line FTC.08 (bar = 25 µm). (C) Action potential width and action potential rise time had different variability for GFP positive and GFP negative control layer 2/3 pyramidal neurons from line FTC.08 (n = 7, 11, respectively). A single action potential was evoked by 5 ms current step to measure the action potential width and action potential rise time GFP positive and GFP negative control layer 2/3 pyramidal neurons from line FTC.08 had similar membrane time constants (37.3±1.4 ms and 36.6±3.2 ms, respectively) and resting membrane potentials (−72.9±2.1 ms and −72.2±1.3 mV, n = 7 and 11, respectively).
(TIF)
