# Supplementary figures and images for: Genetic Labeling of Neuronal Subsets through Enhancer Trapping in Mice
Source: PLoS One. 2012 Jun 7;7(6):e38593. doi: 10.1371/journal.pone.0038593 (PMC3369840; doi:10.1371/journal.pone.0038593)

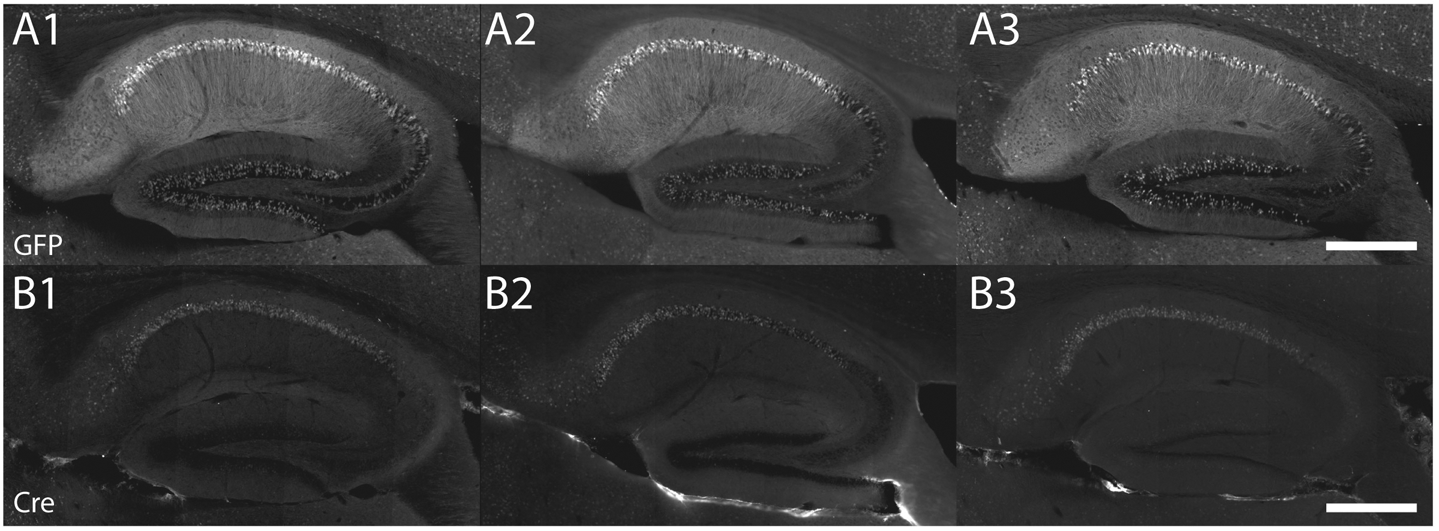

Supplement: Figure S1 — Consistent recombination patterns in transgenic mice carrying a Cre-expressing enhancer probe. (A) Recombination patterns for GFP expression were consistent among animals with the same insertion site of thy1mp-cre. (A1)–(A3) show the dorsal hippocampi of three animals from the FTC.03 line. They shared the same expression pattern (bar = 500 µm). (B) In this transgenic line the persistent cre expression was consistent in three adult animals ((B1)-(B3)). In all animals the persistent cre expression in the adult was much smaller than the density of recombined GFP-positive neurons (bar = 500 um). (TIF) [file pone.0038593.s001.tif]

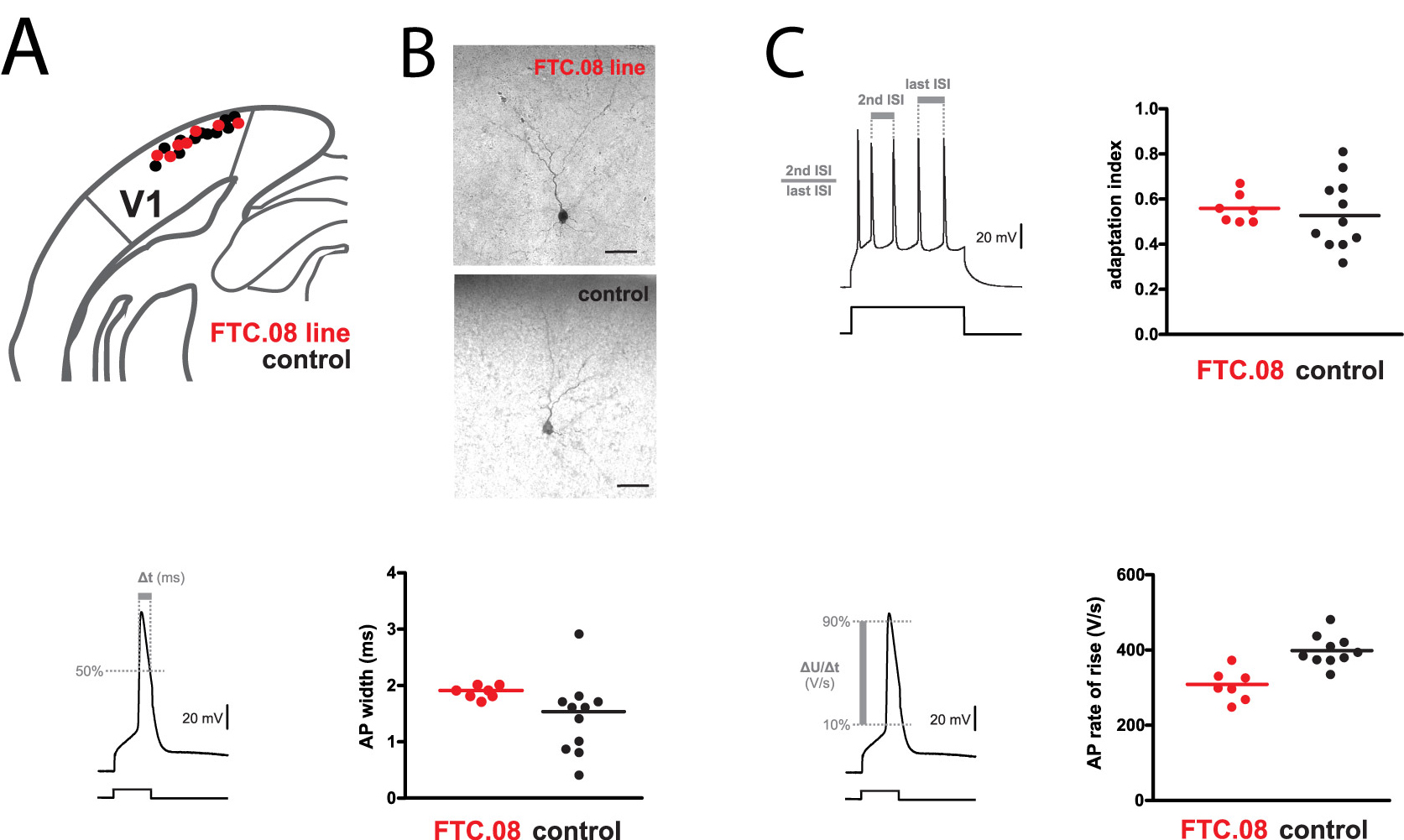

Supplement: Figure S2 — Variability of functional properties in neuronal subsets in thy1mp-cre lines. (A) Recording site in the visual cortex (V1 area) for GFP positive and GFP negative control layer 2/3 pyramidal neurons from line FTC.08. (B) Biocytin-filled GFP positive and GFP negative control layer 2/3 pyramidal neurons from line FTC.08 (bar = 25 µm). (C) Action potential width and action potential rise time had different variability for GFP positive and GFP negative control layer 2/3 pyramidal neurons from line FTC.08 (n = 7, 11, respectively). A single action potential was evoked by 5 ms current step to measure the action potential width and action potential rise time GFP positive and GFP negative control layer 2/3 pyramidal neurons from line FTC.08 had similar membrane time constants (37.3±1.4 ms and 36.6±3.2 ms, respectively) and resting membrane potentials (−72.9±2.1 ms and −72.2±1.3 mV, n = 7 and 11, respectively). (TIF) [file pone.0038593.s002.tif]
